# Economic considerations and health in all policies initiatives: evidence from interviews with key informants in Sweden, Quebec and South Australia

**DOI:** 10.1186/s12889-015-1350-0

**Published:** 2015-02-18

**Authors:** Andrew D Pinto, Agnes Molnar, Ketan Shankardass, Patricia J O’Campo, Ahmed M Bayoumi

**Affiliations:** Department of Family and Community Medicine, St. Michael’s Hospital, 410 Sherbourne Street, 4th Floor, Toronto, Ontario M4X 1K2 Canada; Department of Family and Community Medicine, Faculty of Medicine, University of Toronto, Toronto, Ontario Canada; Centre for Research on Inner City Health, Li Ka Shing Knowledge Institute, St. Michael’s Hospital, Toronto, Ontario Canada; Department of Psychology, Wilfrid Laurier University, Waterloo, Ontario Canada; Dalla Lana School of Public Health, University of Toronto, Toronto, Ontario Canada; Department of Medicine, University of Toronto, Toronto, Ontario Canada; Institute of Health Policy, Management, and Evaluation, University of Toronto, Toronto, Ontario Canada; Division of General Internal Medicine, St. Michael’s Hospital, Toronto, Ontario Canada

**Keywords:** Economic analysis, Healthy public policy, Health impact assessment, Public health, Policy and implementation, Health in all policies

## Abstract

**Background:**

Health in All Policies (HiAP) is a form of intersectoral action that aims to include the promotion of health in government initiatives across sectors. To date, there has been little study of economic considerations within the implementation of HiAP.

**Methods:**

As part of an ongoing program of research on the implementation of HiAP around the world, we examined how economic considerations influence the implementation of HiAP. By economic considerations we mean the cost and financial gain (or loss) of implementing a HiAP process or structure within government, or the cost and financial gain (or loss) of the policies that emerge from such a HiAP process or structure. We examined three jurisdictions: Sweden, Quebec and South Australia. Semi-structured telephone interviews were conducted with 12 to 14 key informants in each jurisdiction. Two investigators separately coded transcripts to identify relevant statements.

**Results:**

Initial readings of transcripts led to the development of a coding framework for statements related to economic considerations. First, economic evaluations of HiAP are viewed as important for prompting HiAP and many forms of economic evaluation were considered. However, economic evaluations were often absent, informal, or incomplete. Second, funding for HiAP initiatives is important, but is less important than a high-level commitment to intersectoral collaboration. Furthermore, having multiple sources of funding of HiAP can be beneficial, if it increases participation across government, but can also be disadvantageous, if it exposes underlying tensions. Third, HiAP can also highlight the challenge of achieving both economic and social objectives.

**Conclusions:**

Our results are useful for elaborating propositions for use in realist multiple explanatory case studies. First, we propose that economic considerations are currently used primarily as a method by health sectors to promote and legitimize HiAP to non-health sectors with the goal of securing resources for HiAP. Second, allocating resources and making funding decisions regarding HiAP are inherently political acts that reflect tensions within government sectors. This study contributes important insights into how intersectoral action works, how economic evaluations of HiAP might be structured, and how economic considerations can be used to both promote HiAP and to present barriers to implementation.

## Background

Several high-profile reports over the past three decades have argued that intersectoral action is required to address the social determinants of health [1-4]. Internationally, governments have taken notice that a comprehensive commitment to health means looking beyond the traditional health care sector. Health in All Policies (HiAP) is a form of such action that seeks to integrate health considerations in the development, implementation and evaluation of policies through conjoint leadership within and across sectors [5,6].

Policymakers have described HiAP implementation as challenging given the need to engage and collaborate with diverse health and non-health sectors [7,8]. Economic considerations may be particularly important for several reasons. By economic considerations we mean the cost and financial gain (or loss) of implementing a HiAP process or structure within government, or the cost and financial gain (or loss) of the policies that emerge from such a HiAP process or structure.

Policy makers with limited time horizons might prioritize short-term economic goals (such as cost control) over long-term health outcomes [9,10]. Yet HiAP interventions may be promoted as a means of controlling long-term health care costs by preventing disease [7]. In addition, intersectoral action is promoted as a way to address overlap and redundancy in times of fiscal constraint [11]. Finally, funding practices, such as integrated budgets and joint accounting, are key for implementing HiAP [8,12]. Policymakers engaged in developing or implementing HiAP initiatives would benefit from a detailed understanding of how economic considerations have been addressed in jurisdictions that have used HiAP.

While a number of descriptions of HiAP implementation have been published, there is little research on how and why such initiatives succeeded or failed in different settings [13]. This study presents a thematic analysis of how economic considerations affected the implementation of HiAP in three jurisdictions. Our objectives were to identify key economic issues and to explore the consistency in how these issues were relevant to implementation in different contexts. By implementation of HiAP processes and the subsequent policies that emerge, we mean actions to carry out governmental decisions as specified through legislation, formal strategy or mandate [14]. We restricted our focus to such actions and did not include implementation related to policy generation or changing governance structures.

## Methods

We conducted an international systematic scoping review in 2011 that identified established, formal HiAP initiatives [15]. HiAP was defined as multisectoral initiatives toward healthy public policy making, coordinated primarily by government, where sectors collaborate by developing policies, programs and projects that include interventions addressing upstream social determinants of health [4,14]. HiAP encompasses both public health interventions and non-health interventions that have health benefits. HiAP objectives have included improving health (and possibly other benefits), establishing the cost-effectiveness of HiAP programs and reducing heath and social inequities. Herein, we focus on questions related to efficiency and equity that arise when implementing HiAP.

From 43 jurisdictions where some form of intersectoral action for health equity had been implemented, we identified 16 that had extensively adopted HiAP policies. Our research team constructed a set of criteria for case selection. We focused on cases where there was a clear mandate for HiAP that could be identified. We included cases that had been initiated within the previous 10 years to ensure that informants could recall past events with sufficient detail, and excluded cases that had been initiated only within the last two years, as they may not yet be viable. We required multiple sources of data on each case, so excluded those with few sources in the grey or peer-reviewed literature. As the focus of this program of research has been implementation, we further excluded cases that had not yet reached the maintenance or evaluation stage. Finally, we chose cases that had similar GDP, similar level of government where HiAP was initiated and implemented and similar policy process. We have completed three explanatory case studies to date – Sweden, Quebec and South Austrialia – that form the basis of this report (Table 1). All cases are high-income countries but represent a variety in terms of the type of welfare state. Ethics approval was obtained from the St. Michael’s Hospital Research Ethics Board.

**Table 1:** **The contextual factors of HiAP implementation in Sweden, Quebec and South Australia**

**Contextual factors**	**Sweden**	**Quebec**	**South Australia**
Welfare state regime	High-income	High-income	High-income
	High in labour market egalitarianism	Low in labour market egalitarianism (Canada)	Low in labour market egalitarianism (Australia)
Mandate type & year	Legislated bill, 2003	Legislated in Public Health Act, 2002	Strategy, 2008
Mandate description	Minister of Health with directors-general of “concerned agencies” guides national, regional and municipal level intersectoral health coordination in regards to the health policy. There was a change in 2008 initiated by the Moderate Party (centre-right) where the focus shifted to elderly, children and young people with emphasis on lifestyle changes.	All prospective policies that may impact population health must pass through a health impact assessment administered by the Ministry of Health and Social Services.	Health lens analyses are used to help government sectors meet targets laid out in the South Australia Strategic Plan (2004). There was a change in 2011 initiated by the Australian Labor Party (left-leaning) with an amendment to the Public Health Act to include a provision that the Minister of Health may provide advice or develop procedures to ensure enhancement of health.
Level of implementation	Country, county, municipal	Province	State, municipal
Financing HiAP	At the national level, there are no financing mechanisms to directly fund sectors to participate in HiAP and to conduct Health Impact Assessments (HIA) but some examples of coordinated budgeting/policy-oriented funding to encourage action on specific objectives. The Swedish National Institute of Public Health provides in-kind support to sectors at all levels of government to integrate health considerations in policymaking, including the use of HIA tools and hosting cross-sectoral meetings. Sectors may pool budgets when they collaborate. At the municipal level HiAP work is financed by the various sectors and from various state/public donors with supported by public health coordinator.	At the provincial level, there are no financing mechanisms to directly fund sectors to participate in HiAP or to carry out HIA. Ministries are expected to provide in-kind support to allow employees to participate in the inter-ministerial committee. The Direction Générale de Santé Publique funds research to support the development of knowledge and expertise in using HIA tools and the Institut National de Santé Publique du Québec provides in-kind support to sectors at all levels government to use HIA tools.	At the state level, there are no financing mechanisms to directly fund sectors to participate in HiAP. Individual projects rely on joint funding from partner agencies. Foundation of HiAP in South Australia acknowledges that many of the most pressing health problems of the population require long-term policy and budgetary commitment, including innovative budgetary approaches. Dedicated funding for global aspects of HiAP initiative provided by South Australia Health, and for dedicated staff in a Health in All Policies Unit provided by Health Promotion and Public Health.

### Key informant recruitment and data collection

We identified potential key informants in each jurisdiction based on a review of grey and peer-reviewed literature. Potential informants were contacted by email or telephone and asked to rate their familiarity with implementation of their specific initiative; those who said they were familiar to very familiar were eligible for an interview. Additional informants were identified through snowball sampling. We conducted semi-structured telephone interviews in the official language of each jurisdiction using a detailed interview guide. Informed consent was obtained from participants and only adults were interviewed. We intervied 12 to 14 persons in each jurisdiction. Further details are available elsewhere[15].

### Data analysis

We used a qualitative iterative approach to develop a thematic analysis of economic considerations [16]. English summaries (Sweden and Quebec) or verbatim transcripts (South Australia) of each interview were read by two members of the investigative team (AP, AM). These investigators independently identified statements relevant to economic considerations and then jointly developed a coding framework based on the health literature and classified statements using this framework. The coding framework included three categories: (1) economic evaluations of HiAP [5,17,18]; (2) resources and financing for HiAP [8,19]; and (3) structural economic considerations (such as the state of the national and international economy and local economic policies) [20-22]. Finally, the entire investigative team reviewed the statements to identify themes. Themes were identified and refined through iterative readings and discussions until no further themes were identified. Important contextual factors were noted. The investigators represent a range of disciplines (medicine, law, health policy, social epidemiology, health economics, public health), thereby enhancing methodological rigor [23].

## Results

Across the three categories, we identified six themes, as described below.

### Economic evaluations of HiAP

#### Economic arguments are important for promoting HiAP

We found general agreement regarding the importance of promoting HiAP based on economic evaluations, meaning the analysis of alternative courses of action in terms of both costs and outcomes [24]. As noted by one informant, “we need to develop more knowledge on these issues in order to meet the demand about which policies are most cost-efficient”. Such evaluations were perceived to facilitate buy-in for HiAP across government sectors. As one informant noted, “it is central today to be able to show that policies are worth it from an economic point of view.” Several informants noted that economic evaluations of HiAP were seen as particularly important for controlling rising health care costs; others argued that HiAP is “efficient” because it represents investing in interventions in order to prevent poor health or social outcomes, which were framed as having a net negative cost to society in the long-term. Thus, HiAP appeared to be seen as providing a means for policymakers to assess the positive and negative health consequences of options. Such consequences could be monetized and the costs or gains could be included in the analysis of options. Yet informants recognized that many governments undervalue preventive services, which may only be considered after the daily “more urgent issues agenda” has been addressed.

#### Many forms of economic evaluation were considered

The specific form of economic evaluation was often not specified but many informants emphasized that HiAP should demonstrate that money will be saved. Others suggested evaluating how much poor population health costs society or focused on the affordability of the HiAP intervention by stressing the direct cost (e.g. staff time, organizing meetings across sectors, etc.) of implementing HiAP. Finally, some suggested that it is important to show that HiAP leads to a “return on investment”, that it was economically efficient, or that it represents the best use of resources compared to alternative courses of action.

#### Economic evaluations of HiAP are often absent, informal or incomplete

The evidence base for economic evaluation was often absent or unclear. Informants described limited experience with economic evaluation, pointing to several administrative and methodological difficulties. For example, the time horizons over which effects could be observed (typically years) are often too long for governments with more immediate concerns. Similarly, the short-term time horizon for implementation costs is often very different from the long-term time horizon for health and social benefits and any attendant savings.

Another important challenge for HiAP is measuring benefits. While health economists have developed methods for incorporating health-related quality-of-life effects, HiAP benefits might include non-health effects that may be difficult to measure and challenging to include on a common scale with health effects. Informants also noted that it could be difficult to attribute changes to HiAP interventions alone given concurrent policy changes.

### Resources and financing for HiAP

The financing of HiAP initiatives varied across cases (Table 1).

#### Funding for HiAP initiatives is important, but is less important than a high-level commitment to intersectoral collaboration

Informants consistently noted that “sometimes, some small amount of additional money is needed [such as] for the organization of intersectoral conferences, but mainly, HiAP does not require more money” and that “some extra money is sometimes useful to get some speed in some projects”. Typically, the financing of HiAP occurred through existing budgets. An informant suggested that, rather than devoting specific resources to the implementation of HiAP, “we should be better at what we are already doing, by thinking strategically about health and sustainable development”. Some informants were concerned that project-specific financing might lead to dis-engagement when funding stops and, therefore, short-lived action.

One informant noted that intersectoral policy work often requires “an extra bit” of effort that can represent considerable uncounted costs. This “in-kind support … is probably the most important source of funding.” That is, “if you put a dollar value and add up all the hours and all the [HiAP] informants who are doing this as an extra bit of their work, you get massive amounts”. As noted by an informant in Sweden, the key objective for those working in small municipalities on HiAP implementation is knowledge sharing rather than gain from new financial resources.

However, an absolute lack of financial resources is a barrier to HiAP implementation. Because financing has symbolic significance, sectors may perceive that a lack of HiAP funding means signals that the initiative is a lower priority. Informants stated that “health is a ‘soft’ issue, when resources are missing, its significance declines” and that “with less resources, people get very much focused on their own mission and they are less open to collaborate intersectorally”. Despite the sense that HiAP could lead to efficiencies and cost-saving in the long-term, another informant suggested that HiAP may not be implemented at all if budgets are too constrained in the short-term.

#### Co-financing of HiAP is seen as being both beneficial and detrimental

Multiple sources of funding, such as pooled budgets across sectors, may support HiAP implementation. As noted by an informant from South Australia, “it’s truly a partnership approach … we’re working together alongside each other to solve and to investigate the problem and to come up with a policy solution.” In Sweden, the central government initiates HiAP projects and ongoing financing and implementation is the responsibility of municipalities. Thus, the two levels of government were reported as “contributing to the financing of the project” and this helped establish both as “co-owners”.

However, several negative aspects of co-financing were identified. It is difficult to assemble resources from different actors that receive financing from different levels of government (municipal, county or regional). An informant from South Australia noted that successfully navigating through different bureaucracies requires a “skill set around engaging with other departments and/or communities.” The need for multiple funding sources can expose underlying tensions between levels of government. For example, key informants reported that municipal governments may bear the burden of HiAP implementation but receive only part of the resources necessary from the central government.

### Structural economic considerations for HiAP

#### The existing tension within government ministries between addressing economic and social/health objectives is highlighted by HiAP initiatives

Some informants identified difficulties in engaging policymakers outside of the health sector in HiAP initiatives. For ministries whose primary goal is economic development, “taking into consideration the health impacts counts for nothing”. An informant noted that when a policy has an impact on “business outcomes … it creates incredible tension … between economic deliverables … and social deliverables”. For example, between increasing employment or economic productive and improving health outcomes. However, policy goals that are framed as having both economic and health or social benefits facilitate buy-in for HiAP. For example, a sustainable development strategy linked to HiAP was highlighted by several informants at the municipal level in Sweden as leading to a high degree of buy-in across sectors. A key scientific report describing the connection between multiple determinants of health and sustainable development, and the integration of public health targets into sectoral plans to achieve sustainable development, attracted a wide range of politicians to participate in the initiative. In addition, inclusion of these targets in the city’s implementation budget signaled that these objectives were political priorities that “automatically” led to the participation of all sectors.

## Discussion

We used thematic analysis to evaluate views regarding economic considerations among stakeholders interested in and participating in implementing HiAP using case studies from Sweden, Quebec, and South Australia. Informants consistently stated that economic considerations are important for promoting HiAP to non-health sectors within governments. This finding itself is not surprising. As others have noted when discussing HiAP, “Health partners must recognize the importance of non-health goals to non-health partners and develop an economic case for action” [8]. However, there was considerable heterogeneity and lack of clarity in how economic considerations were conceptualized. Informants appeared to confuse affordability (i.e. this intervention is possible with our existing resources) with efficiency (i.e. this intervention is a good use of resources, given alternatives).

Economic evaluations of HiAP may provide information that better allows for a more complete cost assessment of different policy and program options. However, few robust economic evaluations of HiAP interventions have been completed to date and there are considerable conceptual and logistical challenges to such evaluations [25-28]. Methodological concerns include questions about the appropriate time horizon for an analysis, the challenges in measuring non-health benefits, and difficulties in comparing benefits on a common scale [9,10]. Costs and outcomes may accrue to different sectors of government, and capturing this data requires extensive cross-sectoral information systems. Existing evaluations on the impact and effectiveness of intersectoral action for health equity also lack descriptions of contextual factors, such as the roles and responsibilities of sectors and intersectoral relationships, and how these were related to observed outcomes of the interventions [29]. The appropriate perspective for the evaluation also needs to be defined. A health system perspective, by definition, would be too narrow for HiAP. A societal perspective, that counts all costs and effects regardless of who pays or benefits, is often favored by economists but might be too broad for governmental decision makers. HiAP economic evaluations will need to develop methods that appropriately reflect a distinct “whole of government” perspective that incorporates relevant trade-offs related to costs and outcomes between sectors.

Many informants felt that demonstrating that HiAP is cost saving would provide strong evidence in favor of sustained implementation. Nevertheless, no informants provided evidence that downstream savings offset the cost of HiAP interventions; instead, such arguments seem to be based on intuitive comparisons of relatively inexpensive implementation costs to the high costs of downstream health and social consequences. Of note, preventative health services are similarly believed to be cost-saving but very few result in net negative costs [30]. Other informants argued that HiAP is worthwhile because the societal cost of continued inaction to address important social determinants, such as poverty, is high. While such arguments might have validity, quantifying the effects of addressing such determinants is beyond the scope of most economic evaluations, which are typically used to guide resource allocation decisions. From this perspective, the most rigorous approach would compare the marginal net cost of a HiAP intervention, relative to a comparator, and the marginal net effects. A few informants framed economic considerations in such terms, using words such as “efficiency” or “cost-effectiveness”.

HiAP is sometimes promoted as a means to reduce health sector costs or as a mechanism for increasing public health funding [7,31]. However, focusing too strongly on the economic outcomes of HiAP might detract from the potential health and social impacts [32]. Furthermore, several informants noted that views of HiAP can change according to economic conditions. Economic downturns and austerity budgets can represent real threats to the implementation of HiAP processes – both because HiAP might be seen as an expendable extra and because sectors become very protective of their own funds and give less priority to intersectoral collaboration [33]. There was a strong sentiment amongst informants that implementing HiAP without dedicated funding left HiAP programs vulnerable to budget cuts.

Few studies have evaluated the costs of implementing HiAP [5]. We note that our informants did not discuss the importance of estimating the cost of scaling up initiatives found to be good value for money. One informant noted that the opportunity cost of HiAP can be considerable if implementation diverts resources from other activities and no extra funding is allocated [34]. Although integrated budgets and joint accounting have been identified as methods to promote the implementation of HiAP [12,19], our results are more equivocal. Multiple sources of funding might be advantageous to generate broader buy-in across levels of government but could be disadvantageous if underlying tensions within government are exposed and exacerbated. Other analyses of HiAP implementation have suggested that leadership, either political or bureaucratic, and fostering personal interactions across networks, is essential to engage diverse stakeholders and manage such tensions [8]. Joint budgeting can be vulnerable to spending cuts as departments often start by reducing or eliminating contribution to intersectoral initiatives [35]. Delegated financing to support HiAP processes are similarly quite susceptible to economic downturns, necessitating creative interventions to keep the public and policymakers supportive of intersectoral action to improve health [8]. Having a specific budget for HiAP can also be a challenge if it reduces the motivation of non-health sector actors to take responsibility for the health impact of their policies [34].

Our study has some limitations. We used a definition of HiAP that focused on health inequities. We recognize that this may have limited the scope of our study and might limit the generalizability of our findings to HiAP interventions where equity is not a primary policy objective. We focused only on three jurisdictions and on implementations that occurred in a particular period. Our coding framework was structured around three categories that developed from initial readings. However, themes could have categorized in a number of different ways. We might have identified additional views regarding implementation and barriers to HiAP in other contexts, beyond the three case studies examined. Particularly important contextual factors might include country, economic conditions, and governmental jurisdictional responsibilities. We also did not explore some macroeconomic issues that might be important for considering HiAP, including tax policies, public and private health insurance systems, and social welfare policies. Finally, our key informants did not mention specific examples or specific policies where economic considerations had particularly been important as they implemented HiAP in their jurisdiction. This lack of concrete examples limits our findings to mostly abstract conceptualizations of how such considerations shape HiAP implementation. Future research on the economic considerations HiAP could explore its application to specific policies, such as those regulating the alcohol and food industry, or economic development and extractive industries.

Ultimately, decisions about which HiAP policies get funded and the amount of resources theyreceive are political decisions that reflect power relations both within and without government, as well as stakeholders’ values and ideological perspectives [8]. As called for in the 2013 Helsinki Statement on Health in All Policies, there is a need for “conflict of interest measures that include effective safeguards to protect policies from distortion by commercial and vested interests and influence” [36] While we focused specifically on economic issues related to the implementation of HiAP interventions, future research should focus on the political economy of implementing HiAP policies.

Our results are useful for elaborating propositions for use in realist multiple explanatory case studies, a new method for understanding how macrosocial health equity interventions are implemented. This approach seeks to explain causal effects within each case by developing a specific understanding of phenomena in relation to the context while refining explanatory theory applicable to the collection of cases by testing main and rival propositions (similar to hypotheses). These propositions are a form of middle-range theory, which fall short of unified theory intending to “explain all observed uniformities of social behavior, social organization and social change” but that can help explain certain occurrences. Propositions are typically developed on the basis of existing theory, past experience and previous evaluations or research studies, including through the observation of semi-predictable, re-occurring patterns identified in data [37].

Accordingly, we suggest two propositions related to economic considerations and HiAP. We stress that these propositions are explanatory rather than normative; that is, they seek to explain how decisions are actually made rather than to propose how they should be made. First, we propose that economic considerations are currently used primarily as a method by health sectors to promote and legitimize HiAP to non-health sectors with the goal of securing resources for HiAP. More specifically, our results suggest that individuals responsible for implementing HiAP are primarily interested in arguments that are focused on seemingly common-sense findings (‘reducing poverty must save money’) rather than on formal analyses, whether simple (such as analyses of implementation costs or budget impact) or complex (such as cost-effectiveness analyses within or across sectors). This proposition diverges from an idealized version of economic evaluation where economic evaluations are used as the primary basis for resource allocation decisions. However, as discussed above, methods to conduct economic evaluations of HiAP initiatives are not well elaborated. Moreover, it is possible that economic arguments could detract from health concerns, perhaps for reasons related to this proposition. Taken together, these considerations suggest that economic evidence is valued for its functional role rather than as intrinsically reflecting the worth of HiAP or for guiding resource allocation decisions.

Our second proposition is that allocating resources and making funding decisions regarding HiAP are inherently political acts that reflect tensions within government sectors. More specifically, governments’ political agendas, the relative power of individual ministries or departments and the state of the economy each influenced whether HiAP was a priority for research allocation and how much funding a specific implementation received. Informants were considerably concerned about whether HiAP funding was secure, whether HiAP resources reflected opportunity costs, and whether HiAP implementation was threatened during times of economic austerity.

HiAP interventions are vulnerable for several reasons. First, HiAP is seen to come from the health sector, which already consumes a large amount of resources. Other sectors might be unenthusiastic about earmarking more funds for health, and evidence shows that even in the case of strong, government wide commitment to HiAP, economic and trade policy considerations can dampen systematic efforts to implement HiAP [38]. As Second, HiAP interventions are sometimes seen as discretionary additions to established systems. Third, cross-jurisdictional funding and responsibility might make HiAP programs particularly vulnerable if they do not have a strong champion [6,39].

## Conclusions

Our propositions contribute to our understanding of HiAP, which currently lacks a strong theoretical base. Our study highlights the need for further work to examine how the successful implementation of HiAP relates to a number of factors in the policy process, one aspect being economic considerations.

Our themes and propositions about the role and use of economic arguments and evaluations in support of HiAP should be examined in additional cases that explore the politics of the budgetary process in-depth, as has been done in other areas [40], and is currently missing from much of the research on intersectoral governance and HiAP. Finally, the specific role of such evidence in ensuring the long-term sustainability of HiAP is also a topic for further inquiry.

## Abbreviations

HiAP: Health in all policies
HIA: Health impact assessment
